# A Biomimetic Multiparametric Assay to Characterise Anti-Amyloid Drugs

**DOI:** 10.3390/ijms242316982

**Published:** 2023-11-30

**Authors:** Willy Smeralda, Marc Since, Sophie Corvaisier, Dimitri Fayolle, Julien Cardin, Sylvain Duprey, Jean-Pierre Jourdan, Christophe Cullin, Aurélie Malzert-Freon

**Affiliations:** 1 Normandie Université, UNICAEN, CERMN, Boulevard Becquerel, 14000 Caen, France; smeralda.willy@hotmail.com (W.S.); sophie.corvaisier@unicaen.fr (S.C.); dimitri.fayolle@unicaen.fr (D.F.); jean-pierre.jourdan2@ght-cdn.fr (J.-P.J.); 2 CIMAP, ENSICAEN, UNICAEN, UMR6252 CNRS, CEA, Normandie Université, 6 Bd du Maréchal Juin, 14050 Caen, France; julien.cardin@ensicaen.fr (J.C.); sylvain.duprey@ensicaen.fr (S.D.); 3 Pharmacie à Usage Intérieur, Centre Hospitalier de Vire, Normandie, 14504 Vire, France; 4 CBMN, CNRS UMR 5248, Université de Bordeaux, 33600 Pessac, France; christophe.cullin@u-bordeaux.fr

**Keywords:** Alzheimer’s disease, liposomes, β-amyloïd, drug discovery, bexarotene, indomethacin, Chicago sky blue

## Abstract

Alzheimer’s disease (AD) is the most widespread form of senile dementia worldwide and represents a leading socioeconomic problem in healthcare. Although it is widely debated, the aggregation of the amyloid β peptide (Aβ) is linked to the onset and progression of this neurodegenerative disease. Molecules capable of interfering with specific steps in the fibrillation process remain of pharmacological interest. To identify such compounds, we have set up a small molecule screening process combining multiple experimental methods (UV and florescence spectrometry, ITC, and ATR-FTIR) to identify and characterise potential modulators of Aβ_1-42_ fibrillation through the description of the biochemical interactions (molecule–membrane Aβ peptide). Three known modulators, namely bexarotene, Chicago sky blue and indomethacin, have been evaluated through this process, and their modulation mechanism in the presence of a biomembrane has been described. Such a well-adapted physico-chemical approach to drug discovery proves to be an undeniable asset for the rapid characterisation of compounds of therapeutic interest for Alzheimer’s disease. This strategy could be adapted and transposed to search for modulators of other amyloids such as tau protein.

## 1. Introduction

The aggregation of amyloid proteins is a key event in neurodegenerative disease pathogenesis, especially in the case of Alzheimer’s disease (AD) [1]. AD is the most widespread form of senile dementia worldwide and represents a leading socioeconomic problem in healthcare [2]. It is characterised by the accumulation of extracellular plaques formed by amyloid-β peptide (Aβ). Although debated, the aggregation of Aβ is linked to the onset and progression of the disease [3].

There are multiple isoforms of the Aβ peptide [4], but in the literature, those that are most currently associated with AD are the Aβ_1-40_ form and the Aβ_1-42_ form. Both result from the β-secretase proteolysis (amyloidogenic pathway) of the transmembrane protein APP present on the surface of neurons [5]. As the disease progresses, dyshomeostasis favours this amyloidogenic pathway, causing the peptides to accumulate in the extracellular space. Once released, the peptides can change their secondary conformation from a random coil to a highly ordered β-sheet structure. This mechanism initiates a process of aggregation into oligomers, protofibrils and mature fibrils, leading to neuronal cell death. As such, the aggregates represent a specific disease marker. Over the last 30 years, the role of these peptides and their aggregated forms in the pathophysiology of the disease has been extensively studied. From the “amyloidgenic hypothesis” point of view, they are considered to be the initiating cause of the disease at two different levels: (*i*) they initiate later events, such as tau protein dysregulation, neuroinflammation, oxidative stress, telomerase inhibition, etc.; and (*ii*) they have a direct cytotoxic action on neurons, especially the Aβ_1-42_ isoform [6].

The mechanisms of the direct cytotoxicity of the Aβ_1-42_ peptides on neurons have been described and explained, particularly in terms of membrane permeabilisation processes. Three mechanisms are described and can mutually cooperate: (*i*) the generation of stable transmembrane toroidal pores permeable to calcium dications, (*ii*) membrane destabilisation via the “carpet model”, and (*iii*) the detergent effect [7].

These direct cytotoxic effects have mainly been characterised by the Aβ_1-42_ peptide in the oligomeric form. In the amyloid aggregation process, oligomers are considered as heterogeneous and unstable intermediates, comprising various types of species, e.g., prefibrillar oligomers, protofibrils, annular protofibrils, paranuclei, globulomers [8], etc. Some can aggregate to form mature fibrils, known as “on-pathways”, while others cannot, known as “off-pathways” [9]. The study of such unstable entities in vitro can be facilitated by their chemical stabilisation [10] or by the use of stable peptide mutants in oligomeric form, such as the oG37C peptide [11,12]. Moreover, this direct cytotoxic effect on the plasma membrane implies the existence of interactions between the peptide and biomembrane, which in turn modulate the aggregation process [13,14,15,16].

A therapeutic strategy proposed by medicinal chemists is the development of molecules able to interfere with specific steps of this fibrillation process [17,18,19,20]. Compounds that would be able to prevent or inhibit the formation of Aβ molecular species that mediate cellular toxicity are particularly appealing [21]. Among these, the development of compounds capable of preventing the formation of toxic oligomers or redirecting aggregation to other pathways is a very attractive approach [22].

In this context, our team has recently developed a study model to explore membrane–Aβ peptide interactions along three axes: membrane permeabilisation, peptide conformational variation, and fibrillation kinetics [12]. This multiparametric characterisation is based on the use of two Aβ peptide forms (commercial wild-type Aβ peptide and the stable oligomeric mutant oG37C) [11,12] and two simple liposomal formulations (phosphatidylcholine, cholesterol and phosphatidylglycerol) mimicking neuronal cell membranes (composition, charge and curvature radius). Some liposomes made of mixtures of soybean phosphatidylcholine (SPC):cholesterol (chol): 1,2-dioleoyl-*sn*-glycero-3-phosphoglycerol sodium salt (DOPG) in a 2-2-6 molar ratio (PCG_226_) appeared to be very appealing to define some specific effects of membranes on the aggregation process: a slowing-down kinetic effect; a promotion of structural polymorphism of Aβ_1-42_, which led to a different oligomer aggregation pathway; and a membrane permeabilisation effect. The use of the second formulation with a molar ratio of 6-2-2 (PCG_622_), which is less rich in negative charges, allows for electrostatic effects to be isolated by comparison.

These effects were measured using simple spectroscopic techniques: (*i*) the kinetics of fibrillisation was monitored by the fluorescence of thioflavine T [20,23], (*ii*) the Aβ peptide secondary structure conformation was characterised by Attenuated Total Reflectance-Fourier Transform InfraRed (ATR-FTIR) spectroscopy [24]; and (*iii*) the impact of Aβ on biomembrane integrity was determined by a liposome leakage assay and dynamic light scattering [25,26].

In the present study, we set up a screening process of small molecules, combining multiple experimental methods to identify and to characterise potential modulators of Aβ_1-42_ fibrillation able to reduce its toxicity through the description of the biochemical interaction of (molecule-membrane-Aβ peptide). The aim of this work is to validate that the proposed model, even if it may be considered simplistic or far from physiological reality, allows for the selection of molecules of interest against Alzheimer’s disease.

The introduction of a small interfering molecule into this binary interaction system requires the description of the other two binary interaction systems. In this way, (molecule–peptide) and (molecule–membrane) interactions can be described using the above-mentioned and complementary techniques to assess the lipophilicity of drug candidates, their impact on membrane fluidity and destabilisation processes associated with membrane toxicity [27,28,29,30].

To establish and validate our multiparametric approach, molecules have been selected based on the reported bibliographic data.

**Figure 1:**
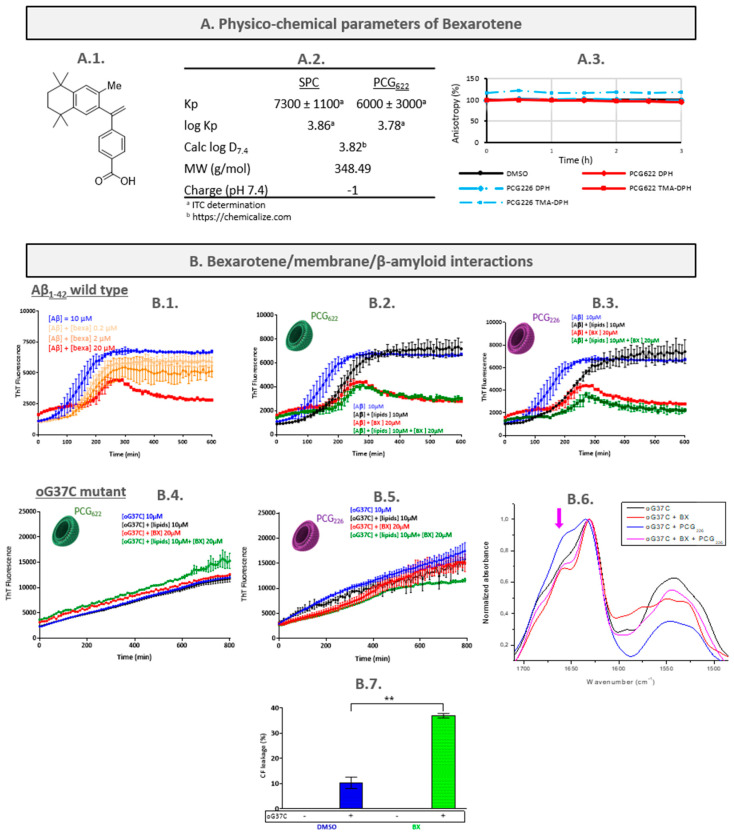
BX characterisation. (**A1**) BX chemical formula; (**A2**) physico-chemical parameters of BX; (**A3**) steady state of diphenylhexatriene (DPH) and its trimethylammonium derivative (TMA-DPH) as a function of time in absence or presence of BX. (**B1**) Aβ_1-42_ wild-type aggregation kinetics with BX at 0, 0.2, 2 and 20 µM; (**B2**) Aβ_1-42_ wild-type aggregation kinetics with BX at 0 and 10 µM in presence or absence of PC_622_LUVs in a molar ratio of 6:2:2 for (SPC):(chol):(DOPG); (**B3**) Aβ_1-42_ wild-type aggregation kinetics with BX at 0 and 10 µM in presence or absence of PC_226_LUVs in a molar ratio of 2:2:6 for (SPC):(chol):(DOPG); (**B4**) oG37C mutant aggregation kinetics with BX at 0 and 20 µM in presence or absence of PCG_622_LUVs; (**B5**) oG37C mutant aggregation kinetics with BX at 0 and 20 µM in presence or absence of PCG_226_LUVs. (**B6**): FTIR spectra of oG37C mutant alone or incubated with BX, PCG_226_ LUVs or both. (**B7**) Carboxyfluorescein leakage from PCG_226_-based LUVs (10 µM) in absence or in presence of oG37C (10 µM), and BX (20 µM) after 4 h incubation at 25 °C. Sample data were analysed by Mann and Whitney statistical test (**: *p* < 0.01).

Bexarotene (BX) is a retinoid X receptor (RXR) agonist and that CSB can mod is now approved by the US Food and Drug Administration and the European Medicines Agency for the treatment of cutaneous T cell lymphoma [31]. This lipophilic molecule shows some structural similarity to the abundant membrane-compound cholesterol (Figure 1A1) [32]. This drug has been reported to reduce amyloid plaques and improve mental functioning in a small sample of mice engineered to exhibit Alzheimer’s disease-like symptoms. It could also stimulate the expression of apolipoprotein E (ApoE), leading to the intracellular clearance of Aβ [33]. In consequence, bexarotene was brought in phase 2 clinical trials to determine its safety profile and its effects on abnormal proteins found in the brain in AD. The conclusion of this trial was negative, with no consistent change in any clinical outcome [34]. However, bexarotene could delay primary nucleation in Aβ_1-42_ aggregation [35,36]. It could also inhibit the production of Aβ by other mechanisms, in particular by inhibiting the intramembrane cleavage of the amyloid precursor protein (APP) by β-secretase [37]. Considering all the suggested pleiotropic effects of bexarotene, with some of them implying membranes, we decided to include bexarotene as a model drug in our study.

**Figure 2:**
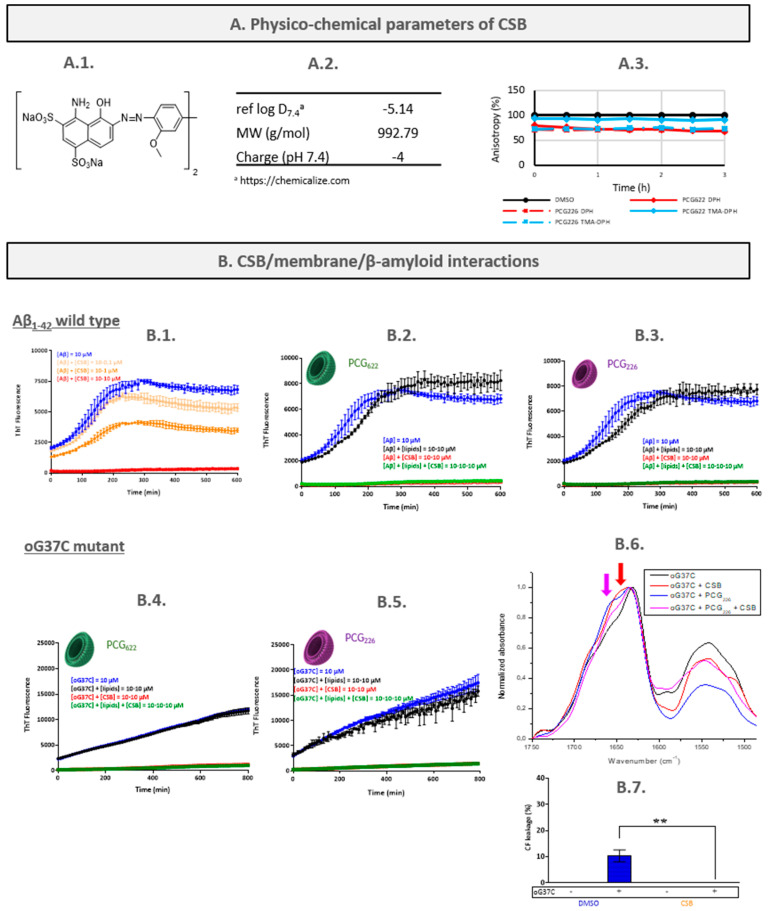
CSB characterisation. (**A1**) CSB chemical formula; (**A2**) physico-chemical parameters of CSB; (**A3**) steady state of DPH and TMA-DPH as a function of time in absence or presence of CSB. (**B1**) Aβ_1-42_ wild-type aggregation kinetics with CSB at 0, 0.1, 1 and 10 µM; (**B2**) Aβ_1-42_ wild-type aggregation kinetics with CSB at 0 and 10 µM in presence or absence of PC622LUVs; (**B3**) Aβ_1-42_ wild-type aggregation kinetics with CSB at 0 and 10 µM in presence or absence of PC_226_LUVs. (**B4**) oG37C mutant aggregation kinetics with CSB at 0 and 10 µM in presence or absence of PC_622_LUVs; (**B5**) oG37C mutant aggregation kinetics with CSB at 0 and 10 µM in presence or absence of PC_226_LUVs. (**B6**) FTIR spectra of oG37C mutant alone or incubated with CSB, PC_226_LUVs or both. (**B7**) Carbo-xyfluorescein leakage from PCG226-based LUVs (10 µM) in absence or in presence of oG37C (10 µM), and CSB (10 µM) after 4 h incubation at 25 °C. Sample data were analysed by Mann and Whitney statistical test (**: *p* < 0.01).

Chicago sky blue 6B (CSB, Figure 2A1) is a counterstain mostly used in immunofluorescence histochemistry for several diagnostic purposes [38]. It is also structurally related to glutamate, which makes it a competitive inhibitor of vesicular glutamate uptake [39,40]. CSB is known as amyloid aggregation modulator with inhibitory activity on α-synuclein, leading to an *in cellulo* neuroprotection effect [41], and with inhibitory activity on the fibrillation of Aβ peptide [42].

Indomethacin is a nonsteroidal anti-inflammatory drug (NSAID) used for its anti-inflammatory, analgesic and antipyretic properties [43]. With regard to the treatment of Alzheimer’s disease, this compound has already been included in a clinical trial to evaluate its ability to slow down the progress of AD, but the results were inconclusive [44]. Indomethacin has been described as a compound able to inhibit Aβ peptide oligomerisation but not its fibrillation [42]. The finding that indomethacin compounds block oligomerisation without inhibiting fibrillation indicates that the inhibited oligomers are not an obligatory step on the pathway leading to fibre formation [42]. The potency to inhibit off-pathway species makes this compound very attractive to be also studied by the multiparametric characterisation tool that we propose.

The present study reports the characterisation of these three molecules using a process combining multiple simple experimental methods. In fine, if validated, this characterisation process could be advantageously proposed in the drug discovery process for AD.

## 2. Results and Discussion

### 2.1. Bexarotene (BX)

#### 2.1.1. Characterisation of the Bexarotene/LUVs Interactions

- Determination of bexarotene partition coefficient

The partition coefficient Kp of bexarotene between biomimetic membranes (i.e., LUVs made either of 100% soybean phosphatidylcholine (SPC), or SPC:cholesterol:1,2-dioleoyl-*sn*-glycero-3-phosphoglycerol in a molar ratio of 6:2:2, PCG_622_) and aqueous buffer was determined by the following: (*i*) a miniaturised and simple UV spectrophotometry method that allows for the rapid determination of partition coefficient values of drugs [27], and (*ii*) isothermal titration calorimetry (ITC), which has been proposed for compounds without chromophoric groups [30]. As no significant spectral shift was observed with increasing lipid concentration, independently of the LUVs composition (Figure S1 for example for PCG_622_-based LUVs), no Kp could be determined for bexarotene using UV-detection. From ITC measurements (Figures S7 and S9), the log $\left(K_{p}^{PCG}\right)$ of 3.86 was determined with SPC LUVs, which is close to the prediction of the log D_7.4_ of 3.82 (Figure 1A2). To our knowledge, it is the first time the partition distribution was experimentally determined for bexarotene. This high value showed that ionised BX at pH 7.4 was very lipophilic and would be mainly inserted in the SPC membrane. In the negatively charged PCG_622_ liposomes, log ($K_{p}^{PCG}$) was somewhat lower at 3.78. This reduction by a factor of two (in absolute terms) was attributed to charge repulsion. To obtain more information about the molecule partition with the LUVs, the bexarotene effect on membrane fluidity was assessed.

- Assessment of bexarotene/membranes interactions by fluorescence anisotropy measurement (FPA)

Interactions between a molecule and membranes can modify the physicochemical properties of membranes, and among them, their fluidity. It can be assessed by measuring changes in fluorescence anisotropy that are the reflection of a probe’s rotational movement caused by stiffness differences in its environment [45]. DPH and TMA-DPH were used as fluorescence probes since the precise positions of these fluorophores along the membrane depth plane are well established. DPH is known to have a deep location and a parallel alignment to the acyl chains. TMA-DPH is a rigid molecule that presents a cationic group attached to the DPH phenyl ring. In the membrane bilayer, due to its amphipathic character, TMA-DPH, with its cationic group, is anchored to the surface of the membrane, i.e., within the polar head groups of the phospholipids, while its DPH phenyl ring is located within the hydrophobic acyl chains of the membrane phospholipids [46].

In comparison with the control (DMSO), the addition of bexarotene caused no significant anisotropy values changes of DPH and TMA-DPH, indicating that there was no major change in membrane fluidity whatever the membrane composition is (Figure 1A3). Although only few articles report bexarotene surface studies, Kamp et al. recently showed that bexarotene was able to be inserted into lipid membranes but did not influence lipid chain order and packing in the membrane [37]. Our results appear consistent with these conclusions.

#### 2.1.2. Characterisation of the Bexarotene/Peptide Interactions

The Aβ peptide aggregation mechanism is characterised by the self-assembly of monomeric species, which is influenced by the presence of several factors until the formation of amyloid fibres [47,48]. This process implies the formation of soluble intermediate species, including oligomeric and protofibrillar species leading to the formation of fibres [49]. The variability of the factors that influence the kinetics of aggregation of the peptide as well as the polymorphism of the species formed have led to divergent aggregation models [50,51,52,53]. On- and off-pathway paradigms have been proposed [11,24,54].

The amyloid formation has been investigated in a Phosphate-buffered Saline (PBS) solution (pH 7.4) containing 10 µM recombinant Aβ_1-42_ peptide, increasing concentrations of bexarotene ranging from 0 to 20 µM, and thioflavin T (ThT).

The aggregation of the peptide alone follows three phases: lag, elongation and plateau (Figure 1B1). This fibrillation process appeared to be affected by the presence of bexarotene, in a concentration-dependent manner: after a lag phase (corresponding to nucleus formation) that lengthens with increasing BX concentrations, exponential aggregation was observed. After 250 min, a plateau was reached with a steep decrease in fluorescence intensity values at the highest tested BX concentration (20 µM). These results indicate that bexarotene can delay the formation of nuclei and oligomers, and thus inhibit the formation of fibrils. Habchi et al. reported that bexarotene selectively targets the primary nucleation step in Aβ_1-42_ aggregation, resulting in a delay in peptide fibrillation [35]. Huy et al. also showed that bexarotene prolongs the lag phase without being able to reduce amyloid deposits [36].

#### 2.1.3. Characterisation of the Peptide/Bexarotene/LUVs Interactions

- Aβ_1-42_/bexarotene/LUVs interactions

As determined previously [12], in the presence of PCG_622_-based LUVs (10 µM), the lag phase increased to about 100 min vs. 60 min for the Aβ_1-42_ peptide used alone (Figure 1B1). This delay in the aggregation process shows that interactions between membranes and Aβ influence the nucleation process of the peptide when used in a 1:1 molar ratio. However, no major quantitative difference was observed at the plateau.

The superposition of curves obtained for Aβ_1-42_/bexarotene with or without PCG_622_-based LUVs evidenced that the effect of the drug on Aβ_1-42_ aggregation was not modified by the presence of membranes. Indeed, when bexarotene was simultaneously added to LUVs in a lipid/drug/Aβ molar ratio of 1:2:1, the interactions between bexarotene and the peptide became predominant and directly influenced the fibrillation process. The influence of the membrane may be modulated by increasing the LUVs relative concentration (Figure S2). Moreover, as observed by using a second formulation of LUVs (SPC:cholesterol:DOPG in a molar ratio of 2:2:6, and named PCG_226_), the nature of the phospholipid head groups, and hence the surface characteristic of the membranes, both modulate the Aβ aggregation pattern. Interactions between the LUVs and the peptide decreased with increased electrostatic repulsions (Figure 1B3). This observation was independent of the lipid/drug/Aβ molar ratio with PCG_226_ (Figure S2). In the presence of these LUVS, the influence of bexarotene on the Aβ aggregation pattern appeared to be predominant. From the Kp value obtained, it is possible to calculate the mol% of BX that is free in the solution or bound to liposomes (Equation (S6)). PCG_622_-LUV could only trap a maximum of 5.1% at 10 µM.

Thus, depending on the concentration of bexarotene used, and the membrane model or the cellular type implied in assays, the interactions between bexarotene and Aβ may be misevaluated. They can then lead to different conclusions [55]. However, from our studies, it is obvious that bexarotene delays the fibril growth, by interfering with Aβ species present at the early stages of the fibrillation process. Such effects of bexarotene on the prolongation of the lag phase are consistent with those already reported in the literature [20,36]. Because oligomers are considered the most toxic Aβ intermediate species, the interactions were further studied by using an oligomer model [56,57].

- oG37C/bexarotene/LUVs interactions

The aim of our screening process is to identify modulators of the aggregation process that are able to reduce Aβ toxicity especially in its oligomeric forms. oG37C is a mutant of Aβ_1-42_ peptide [11] showing a stable oligomeric form associated with high membrane toxicity [26]. It is used here as an Aβ oligomer model [12]. Before this work, oG37C peptide was never evaluated in the presence of a small molecule aggregation modulator.

In the ThT fluorescence assay, oG37C showed a characteristic linear aggregation kinetics throughout 800 min of incubation time (Figure 1B4). This is consistent with the description of Bobo et al. [58]. In their study, they showed that, in the presence of 1,4-dithiothreitol (DTT), it took 960 min to observe the appearance of a sigmoid curve characteristic of amyloid aggregation. In the present study, considering the lower concentration of oG37C (10 μM vs. 30 µM) and the lower temperature (25 °C vs. 30 °C) used, slower kinetics are expected, as it allows us to focus on the early interactions between oligomer molecules.

oG37C aggregation appeared to be only slightly influenced by the presence of bexarotene at 20 µM, and depending on to the experiment, was accompanied by either a slight increase or decrease in ThT fluorescence. When PCG_622_- or PCG_226_-based LUVs were added, no significant modification in the ThT fluorescence kinetics was observed (Figure 1B4,B5). Next, we analysed the interactions with the PCG_226_ model, considering that oG37C preferentially interacts with DOPG through electrostatic interactions with the phosphatidyl group, and can destabilise liposomes made of DOPG [26,58].

Using ATR-FTIR spectroscopy, the oG37C/bexarotene/PCG_226_-based LUVs interactions were studied according to a second parameter: the peptide secondary structure. As shown in our previous work [12], the conformation of the oG37C peptide alone (Figure 1B6, black curve) results in four peaks (1619–1629, 1648, 1665 and 1683–1690 cm^−1^), indicating the presence of β-sheet conformations, α-helix/random, β-turns and anti-parallel β-sheets, respectively [11].

In the presence of liposomes, a strong intensity increase was observed at 1665 cm^−1^. The presence of lipids seems to destabilise the β-sheet structuration, creating an overlap between the β-turn and the α-helix/random band (Figure 1B6 blue curve, pink arrow). In presence of bexarotene alone (Figure 1B6 red curve), the three same peaks were identifiable, but were better defined with less overlap between them. Bexarotene therefore seems to promote structural reorganisation of the peptide. BX showed the same effect in the presence of PCG_226_-based LUVs, but the structuring process was less important.

In summary, the presence of PCG_226_-based LUVs destabilises oligomers, whereas bexarotene promotes their structuring. When all these molecular actors are brought together, the effect of bexarotene with the peptide appears to be predominant (Figure 1B6, pink curve).

To complete the interaction study, we examined the effects of bexarotene and oG37C on membrane permeability using a liposome leakage assay (LLA). PCG_226_-based LUVs were formulated with a self-quenching concentration of carboxyfluorescein (CF). In this assay, a change in membrane permeability causes the self-quenched CF to leak out of the lipid vesicle into the external compartment where the fluorophore becomes highly fluorescent [12,27,59]. The fluorescence increase over time is indicative of heightened membrane permeability. After the completion of the experiment, Triton X-100 was mixed with the vesicles to disrupt them and determine the maximum percentage of leakage.

The exposure of PCG_226_-based LUVs to oG37C in a 1:1 lipid/peptide molar ratio led to about 10.3 ± 2.3% CF leakage (Figure 1B7). Bexarotene alone did not induce any release of CF, but significantly increased the membrane permeabilisation effect of oG37C, with 37.0 ± 1.0% release when using 1:1:2 lipid/peptide/BX. This result was confirmed by DLS; after 24 h incubation in both conditions, LUVs were mostly destroyed and reorganised (Table S1A). This difference in % CF leakage could be due to the formation of differently structured species due to the detergent effect of the combination of bexarotene and oG37C, causing greater disorganisation of the liposomes. Using a PCG_622_-based LUV formulation that is less rich in negative charges, no CF leakage was detected, whatever the conditions were. Thus, this permeabilisation effect seemed to be directed by electrostatic interactions.

- BX mechanism hypothesis and discussion

Bexarotene could influence the nucleation phase (lag phase) of the aggregation process by slowing it down, but the presence of liposomes could attenuate this effect. Bexarotene also appeared to modify the nature of oG37C oligomers, resulting in increased liposome toxicity.

By acting on the early species (nuclei and oligomers), bexarotene could lead to the formation of different oligomeric species via different aggregation pathways as demonstrated by the example of the oG37C. The balance between the different aggregation pathways could be decisive for the ability of bexarotene to reduce the formation of certain species compared to others, all these processes being influenced by external parameters such as the presence of membranes. So far, in the literature, the most studied approach has remained the enhancement of Aβ clearance by apoE activation by bexarotene [33,55]. But according to studies by Ghosal et al., relative to a phase Ib proof of mechanism trial, bexarotene would increase apoE levels in CSF without any effect on Aβ metabolism [60]. This wide variability in effects (and not all of them would likely be known so far) might explain the difficulty in using bexarotene therapeutically directly as a modulator of peptide aggregation.

Although the interpretation of the data obtained with bexarotene may appear complex, the membrane permeabilisation effect observed in the presence of oG37C is a drastic selection element in our process. As a result, bexarotene would not be selected for future studies.

### 2.2. Chicago Sky Blue 6B (CSB)

#### 2.2.1. Characterisation of the CSB/LUVs Interactions

- Determination of CSB partition coefficient

In the presence of SPC or PCG_622_-based LUVs, no significant spectral shift was observed when the lipid concentration increased. Thus, no $K_{p}$ could be determined for CSB. However, this result was predictable as CSB is a highly polar compound with a predicted log D_7.4_ value of −5.14 (Figure 2A2).

- Assessment of CSB/membranes interactions by fluorescence anisotropy measurement (FPA)

In the presence of CSB, a slight decrease in DPH anisotropy was observed for every studied LUV model, whereas a slight decrease in TMA-DPH anisotropy was shown only with PCG_226_-based LUVs (Figure 2A3). Due to the extremely high ionisation charge of CSB, there is a very low probability of CSB interacting with the acyl chains of the membrane. Knowing the fluorescence quenching properties of CSB, spectroscopic artifacts between Chicago sky blue and DPH can explain this result.

No significant membrane-disruptive effect due to CSB was observed on PCG_226_-based LUVs (Figure 2B7), and from DLS measurements performed on these samples at the end of experiments, all the LUVs remained intact after incubation with the molecule for 24 h (Table S1B). In conclusion, it appears that no or only very weak interactions between CSB and model membranes exist.

#### 2.2.2. Characterisation of the CSB/Peptide Interactions

In the presence of CSB, a concentration-dependent decrease in the ThT fluorescence signal at the plateau was observed with a huge effect at the highest tested concentration (10 µM) (Figure 2B1). CSB is known to reduce ThT, emission at such a high concentration, making the ThT assay tricky to assess its role on amyloid formation. Nevertheless, normalised curves exhibit the same characteristics. This is consistent with the conclusions of Necula et al. [42], who also reported the ability of CSB to inhibit Aβ_1-42_ fibrillation, using ThT fluorescence, light scattering and TEM (Transmission Electron Microscopy) analysis.

#### 2.2.3. Characterisation of the Peptide/CSB/LUVs Interactions

- Aβ_1-42_/CSB/LUVs interactions

The tests performed with Tht fluorescence and CSB at high concentration appeared to have low interest. At 10 µM the quenching was so strong that it is more related to a fluorescence quenching effect related to the absorption spectrum of CSB [42]. In this case, all the results presented in Figure 2B2–B5 were not interpretable. At lower concentrations (0.1–1 µM, Figure S3), in the presence of 100 µM lipid, a combined kinetic and quantitative effect on the plateau phase has been observed due to lipids and CSB, respectively, confirming our previous results.

- oG37C/CSB/LUVs interactions

Under these conditions, ATR-FTIR is an alternative technique for the study of peptide secondary structure changes. In the presence of CSB, there was a large broad band between 1629 and 1648 cm^−1^ (Figure 2B6, red arrow) reflecting a higher proportion of the α-helix/random conformation with a lower abundance of β-turns at 1666 cm^−1^. Then, CSB appeared to induce the formation of differently structured species. This reorganisation could explain the fibrillation inhibition observed in the ThT assay.

In the presence of CSB and PCG_226_-based LUVs, an intermediate effect was observed. There was a lower increase in the overlapped α-helix/random and β-sheets signals (Figure 2B6, pink curve) and a lower anti-parallel β-sheet signal compared to oG37C and CSB without lipids. The presence of CSB seems to lead to the formation of species that may be different than the one formed in absence of CSB. It is also possible that these species are formed in the absence of CSB but in smaller quantities.

A protective effect of CSB was shown by LLA experiments. Indeed, no leakage of liposomes occurred in the presence of CSB and oG37C (Figure 2B7), whereas oG37C alone induced significant LUV toxicity. This was also confirmed by DLS experiments (Table S1B). It was observed that a higher proportion (~50%) of the LUVs remained totally intact after incubation with oG37C in the presence of CSB for 24 h. Thus, CSB would not only interact with oG37C but would also limit its toxicity towards the membranes.

- CSB mechanism hypothesis and discussion

In reported studies, CSB has been defined as a compound only able to inhibit the fibrillation of Aβ peptide but not oligomerisation [42]. Our results are not contradictory because they showed that CSB can modify the structuration and toxicity of oligomeric species already present in solution but did not allow for the conclusion of a potential inhibition of their formation. The highlighted protective effect of CSB on Aβ was already objectified by in vitro cellular assays [61]. All results revealed the potential of CSB as a compound of interest for the development of a therapeutic approach. However, as CSB has been previously shown to inhibit the DNA recombinase Rad51 and the glutamate uptake in the synaptic vesicles [62], so it would be difficult to further carry this drug into clinics.

With compounds such as CSB that may induce artifacts in some type of experiments (i.e., fluorescence quenching in the ThT assay), the proposed multiparametric workflow has helped to alleviate doubts thanks to multiple experiments with correlated results. In addition, with regard to the ATR-FTIR study, it would be interesting to find a molecule with the same effect on the peptide signature to see if there is a correlation with reduced toxicity.

### 2.3. Indomethacin (IND)

#### 2.3.1. Characterisation of the IND/LUVs Interactions

- Determination of IND partition coefficient

The partition coefficient of indomethacin was found to be equal to 3.09 or 2.75 on SPC-based LUVs using UV detection or ITC methodology (Figures S5, S6 and S8). With PCG_622_ LUVs, only ITC provided interpretable results, with an approximately two-fold decrease in membrane partitioning ($log\left(K_{p}^{PCG}\right)$ = 2.56) compared to SPC LUVs. These results are very different from the calculated value of log D_7.4_ = 0.68. but are in line with other studies that have shown significant interaction between IND and biomembranes. [29,63]. The results would therefore support the value of using liposomes in the characterisation of interactions between molecules and membranes.

- Assessment of IND/membranes interactions by fluorescence anisotropy measurement (FPA)

**Figure 3:**
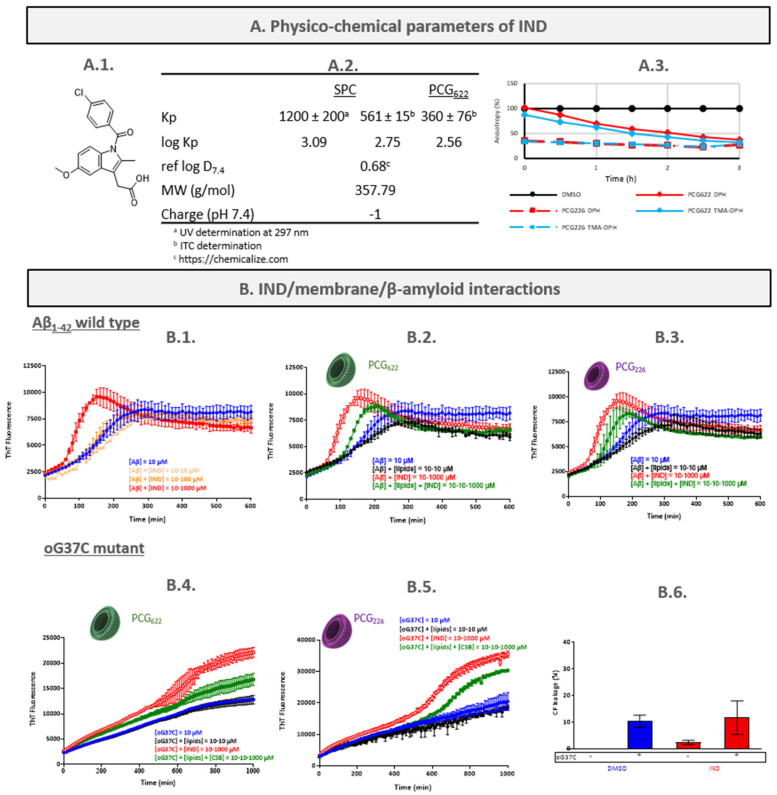
IND characterisation. (**A1**) IND chemical formula; (**A2**) physico-chemical parameters of IND; (**A3**) steady state of DPH and TMA-DPH as a function of time in absence or presence of IND. (**B1**) Aβ_1-42_ wild-type aggregation kinetics with IND at 0, 10, 100 and 1000 µM; (**B2**) Aβ_1-42_ wild-type aggregation kinetics with IND at 0 and 1000 µM in presence or absence of PC_622_ LUVs; (**B3**) Aβ_1-42_ wild-type aggregation kinetics with IND at 0 and 1000 µM in presence or absence of PC_226_LUVs. (**B4**) oG37C mutant aggregation kinetics with IND at 0 and 1000 µM in presence or absence of PC622 LUVs; (**B5**) oG37C mutant aggregation kinetics with IND at 0 and 1000 µM in presence or absence of PC_226_ LUVs. (**B6**) carboxyfluorescein leakage from PCG_226_-based LUVs (10 µM) in absence or in presence of oG37C (10 µM), and IND (1000 µM) after 4 h incubation at 25 °C. Sample data were analysed by Mann and Whitney statistical test.

In the presence of indomethacin, changes in DPH and TMA-DPH were observed for every type of tested LUV (Figure 3A3). In the presence of PCG_622_-based LUVs, there was a time-dependent decrease in both DPH and TMA-DPH anisotropy. When indomethacin was incubated with PCG_226_-based LUVs, an immediate decrease in DPH and TMA-DPH anisotropy was observed. Indomethacin should be anchored within the membrane and would enhance the fluidity of the tested liposomes LUVs. Depending on the liposome composition, the incorporation of indomethacin has led to interactions and membrane reorganisation, either immediately or slowed down over time. Zhou et al. [64] examined the ability of indomethacin to alter membrane heterogeneity and phase behaviour by fluorescence lifetime imaging, fluorescence polarisation and FRET approaches. They showed that indomethacin can enhance phase separation and stabilise cholesterol-dependent nanoclusters in biological membranes [64]. Fearon and Stokes showed that indomethacin can concentrate in lipid bilayers where gel and fluid domains coexist [65]. Nunes et al. showed that indomethacin may be located in the higher-ordered regions close to the phospholipid head groups (C1–C9) at pH 7.4 [63]. This result is consistent with the effects that have been observed in the present study, both on DPH and TMA-DPH probes. These data taken together highlight the fact that indomethacin may interact with membranes in several ways depending on the physicochemical parameters, like the lipid phase, but also on the presence of clusters linked to the presence of cholesterol.

#### 2.3.2. Characterisation of the IND/Peptide Interactions

According to Figure 3B1, no effect was detected on Aβ_1-42_ aggregation using 10 and 100 µM of indomethacin. At the highest tested concentration of 1 mM, an acceleration of the lag phase and the elongation process was observed. Instead of reaching the kinetic plateau in 300 min (Aβ_1-42_ alone) the plateau was reached in 100 min. This result can be explained by the inhibition of the intermediate species formation. By blocking off-pathway oligomerisation without inhibiting fibrillation [42], indomethacin would induce an aggregation kinetics acceleration (off-pathway being a kinetic trap) without impacting the fibrillation plateau.

#### 2.3.3. Characterisation of the Peptide/IND/LUVs Interactions

- Aβ_1-42_/IND/LUVs interactions

In the presence of each biomembrane, only the highest concentration of indomethacin (1 mM) led to an effect like the one observed in absence of liposomes, i.e., fibrillation acceleration (Figure 3B2). The indomethacin effect was in opposition to what was observed with liposomes alone, which caused a fibrillation delay (300 min to reach the plateau for Aβ_1-42_/PCG_622_-based LUVs vs. 200 min without liposomes). The acceleration of the fibrillation by indomethacin was less important in the presence of LUVs (200 min to reach the plateau for Aβ_1-42_/PCG_622_-based LUVs/indomethacin vs. 150 min without liposomes), which was therefore probably due to a combined effect of indomethacin and liposomes on peptide aggregation. These observations suggest interactions between the peptide and the molecule also implying lipids. Such interactions appear independently of the lipid concentration (10 or 100 µM, Figure S4).

- oG37C/IND/LUVs interactions

From Figure 3B4,B5, oG37C aggregation was modified by the presence of indomethacin at 1 mM. The ThT signal of oG37C aggregation kinetic took the shape of a sigmoidal curve with a lag phase, an elongation, and a saturation-phase characteristic of amyloid aggregation instead of the expected linear aggregation kinetics. Another aggregation pathway would be created or amplified in the presence of indomethacin and allow for the early formation of fibrillary species during the aggregation of oG37C. As observed for Aβ_1-42_, the impact of indomethacin was retained in the presence of LUVS, more particularly with those based on PCG_226_.

An ATR-FTIR conformation study would be relevant here. However, the high concentration of indomethacin (1 mM) prevented the isolation of the peptide signal.

Although indomethacin interacts with the membranes, the drug did not exert any significant membrane-disruptive effect as shown by LLA (Figure 3B6) and DLS measurements (Table S1C). No significant difference in leakage was observed between oG37C alone (~10%) and oG37C in the presence of indomethacin (~11%). However, after 24 h, DLS experiments showed a surprising membrane reorganisation effect with the formation of a significant population (~46%) of smaller particles with a mean diameter of 49.6 nm.

Among the mechanisms that have been suggested to describe interactions between Aβ peptide and membranes, there are the covering of the membrane (carpet effect), the permeation of the membrane (pore formation), and membrane dissolution (detergent effect) [66]. The appearance of smaller particles in the presence of both indomethacin and oG37C is caused by a detergent effect. This is facilitated by the previously observed increase in membrane fluidity in the presence of indomethacin.

Thus, indomethacin showed the ability to promote oG37C fibrillation. With this organisational change, the peptide could have an increased affinity for the membrane. When oG37C species come at the vicinity of PCG_226_-based LUVs, the peptide would quickly diffuse and insert into the membrane. Although the membrane was broken, oG37C, due to its hydrophobicity increased by its new conformation, would remain associated with the phospholipids. Smaller vesicles would appear, corresponding to those measured in DLS. Such membrane reorganisation upon oG37C has been already hypothesised from high-speed atomic force microscopy pictures using a slightly different membrane composition (sphingomyelin/PC/GM1 ganglioside/chol) [67]. This is the first time that it is described and correlated to the effect of a drug, namely indomethacin.

- IND mechanism hypothesis and discussion

The ability of indomethacin to promote fibrillation has been established, irrespective of the peptide and liposomal model used, and associated with lipid reorganisation due to the detergent effect in the presence of oG37C.

It should be noted that to observe the effects of indomethacin, very high concentrations (100-fold greater than peptide concentration in our case) would be required, making this drug not an ideal candidate for clinical tests or even in vivo studies. However, as an in vitro model molecule, indomethacin should provide valuable information for understanding the process of peptide aggregation and the advancement of the the field of drug discovery. The effect observed on oG37C with IND, although not showing any protective effect on liposomes, seems to be of interest as a modulator for Aβ aggregation.

## 3. Materials and Methods

### 3.1. Materials

Soybean phosphatidylcholine (SPC, Lipoid S100); 1,2-dioleoyl-sn-glycero-3-phosphoglycerol; and sodium salt (DOPG) were gifts from Lipoid GmbH (Ludwigshafen, Germany). 3-(trimethylsilyl) propionic-2,2,3,3-d4 acid (TMSP); bexarotene; carboxyfluorescein (CF); cholesterol; Chicago sky blue 6B; 1,6-diphenyl-1,3,5-hexatriene (DPH); dimethyl sulfoxide (DMSO); potassium chloride; potassium phosphate; Sepharose^®^ CL-4B; sodium chloride; sodium phosphate; thioflavin T; TMA-DPH (1-(4-trimethylammoniumphenyl)-6-phenyl-1,3,5-hexatriene p-toluenesulfonate); and Triton X-100 were purchased from Sigma-Aldrich (Saint-Quentin Fallavier, France). Ammonium hydroxide was obtained from VWR (Randor, PA, USA), dithiothreitol from Thermo Fisher Scientific (Waltham, MA, USA), and Hepes buffer from Grosseron (Couëron, France). Indomethacin was purchased from Alfa Aesar (Thermo Fisher Scientific, Illkirch, France). Recombinant Aβ was purchase from rPeptide (Wattkinsville, GA, USA)

### 3.2. Production and Purification of oG37C

The oG37C variant was produced and purified with minor modifications of the protocol previously described [11]. Briefly, after transformation in the bacterial strain 969 (Bl21de3pLysS) with the plasmid pET Sac Abeta, one clone was grown on 10 mL LB medium preculture that contained 1% dextrose, 100 mg/L ampicillin and 25 mg/L chloramphenicol. This one-day preculture was added to 990 mL of ZYM medium (1% Tryptone, 0.5% yeast extract, 25 mM Na_2_HPO_4_, 25 mM KH_2_PO_4_, 50 mM NH_4_Cl, 5 mM Na_2_SO_4_ and 2 mM MgSO_4_) containing 100 mg/mL ampicillin and 25 mg/mL chloramphenicol and incubated overnight at 37 °C. After lysis, inclusion bodies were solubilised at 1.25 mg per 10 mL of TE-urea buffer (8 M urea, 50 mM Tris, 1 mM EDTA pH 8). After being left overnight at 4 °C with gentle agitation, the soluble inclusion bodies were centrifuged for 30 min at 30,000× *g* at 4 °C. The supernatant was passed through a 30 kD filtration unit, and monomeric and oligomeric species of Aß were separated by size-exclusion chromatography using a Superdex-75 10/300 GL Column equilibrated in phosphate-buffered saline (137 mM NaCl, 2.7 mM KCl, 10 mM Na_2_HPO_4_ and 1.76 mM KH_2_PO_4_, pH 7.4) at 4 °C. The fractions were pooled separately, and aliquots were quantified using the Bradford assay, frozen in liquid nitrogen and conserved at −80 °C until use.

### 3.3. Liposomes

#### 3.3.1. Formulation of Liposomes

Various liposome compositions were used in this study, based on either 100% SPC, or on mixtures of SPC:cholesterol:DOPG in a molar ratio of 6:2:2 (PCG_622_) or 2:2:6 (PCG_226_). Liposomes were formulated according to the adapted method of the thin lipid-film hydration [68]. Lipid solutions in chloroform/methanol (4:1) were evaporated under nitrogen flow and left under vacuum for 3–4 h to form a lipid film. This thin lipid film was then hydrated in Hepes buffer (Hepes 50 mM, NaCl 107 mM, pH 7.4) for liposomes dedicated to partition coefficient determination or Phosphate-buffered Saline (PBS) buffer (137 mM NaCl, 2.7 mM KCl, 10 mM Na_2_HPO_4_, and 2 mM KH_2_PO_4_) for other experiments and vortexed. Rehydrated lipid suspensions were subjected to 1 h of gentle agitation to have maximum homogenisation. Freeze–thaw cycles were carried out with liquid nitrogen and a 40 °C water bath. The yielded multilamellar vesicles (MLVs) were then extruded 13 times with a mini extruder (Avanti Polar Lipids, Inc., Alabaster, AL, USA) through polycarbonate membranes with a pore diameter of 100 nm (Avanti Polar Lipids, Inc.) to form LUVs.

#### 3.3.2. Characterisation of Liposomes by Dynamic Light Scattering

The average hydrodynamic diameter associated with the polydispersity index (PdI) of the formulated LUVs were measured by dynamic light scattering (DLS) using a NanoZS^®^ apparatus (Malvern Instruments SA, Worcestershire, UK). The zeta potential was calculated from the electrophoretic mobility using the Smoluchowski equation, also using a NanoZS^®^ apparatus. The measurements were performed in triplicate at 25 °C after a 1:100 (*v*/*v*) dilution in the buffer.

### 3.4. Lipid Quantification by NMR Spectroscopy

The lipid concentration of the formulated LUVs was quantified by ^1^H NMR spectroscopy [69]. Briefly, ^1^H NMR measurements with continuous-wave water presaturation were performed in deuterated methanol using TMSP (2 mM) as an internal standard. The CH3 peaks of acyl chains and cholesterol, where applicable, were integrated against the Si(CH3)3 signal of TMSP.

### 3.5. Determination of Partition Coefficient by Derivative UV-Spectrophotometry

The partition coefficients (Kp) of the molecules were determined according to our previous methodology [27]. Briefly, 5 µL of each stock compound solution (750 µM in DMSO) was added to Hepes buffer (50 mM, 107 mM NaCl, pH 7.4) with increasing concentrations of phospholipids (0 to 900, 1000 or 4000 µM) to give a final volume of 250 µL. The corresponding reference solutions were identically prepared in the absence of the molecule. The microplate was incubated in a Biotek Synergy 2 microplate reader (Biotek, Colmar, France) at 37.0 °C ± 0.1 °C for 1 h with regular homogenisation. The absorbance spectra were then recorded using a 1 nm wavelength interval in the 250–500 nm range at 37 °C. The corrected absorption spectra of compounds were obtained by subtracting the spectrum of the liposomal solution used at the same concentration. Spectra data recovery was performed using Microsoft^®^ Excel^®^ 2016. Second derivative spectra were calculated by using a second-order polynomial convolution of 9 points with GraphPad Prism (version 6.01, GraphPad Software, La Jolla, CA, USA). The following equation was applied to determine the *K_p_* value after a non-linear regression analysis and a graph plotting performed with GraphPad Prism:

(1) $$D_{t}=D_{W}+(D_{L}-D_{W})\frac{K_{p}\gamma [L]}{1+K_{p}\gamma [L]}$$

where *D_t_* is the derivative value of the absorbance spectrum of the molecule at a given λ and a given concentration of phospholipids, *D_L_* corresponds to the derivative value of the absorbance spectrum of the molecule in the lipid phase, *D_W_* is the derivative value of the absorbance spectrum of the molecule in the aqueous phase, and [*L*] is the experimental concentration in phospholipids. *γ* is the phospholipid molar volume (considered equal to 0.70 L·mol^−1^ for all experiments). All experiments were performed in triplicate, by using 3 measurements each time. Data are presented as means ± standard deviation of the three experiments.

### 3.6. Determination of Partition Coefficient by Isothermal Titration Calorimetry (ITC)

Large unilamellar vesicles (LUVs) for isothermal titration calorimetry were prepared in a “matched buffer” composed of 50 mM Na-Hepes at pH 7.4, 107 mM NaCl and 2 vol% DMSO to match the injection solutions, following the protocol above. ITC was performed using a Malvern Microcal PeaQ apparatus (Malvern Instruments SA, Worcestershire, UK), equipped with a 270 µL overflowing coin-shaped measurement cell. The titration chamber was thermostated at 25.0 °C throughout the experiment, and the reference cell was heated at a constant power of 5.00 µcal/s. After an initial 0.4 µL dummy injection, 18 injections of 2 µL were performed with 150 s intervals. Stock solutions of the drug compounds were prepared in DMSO at the appropriate concentrations and diluted 50-fold with Hepes-NaCl buffer just before injection to yield the same final buffer composition as the matched buffer used to prepare the LUVs. LUVs were used undiluted.

To determine Kp, a reverse titration was performed. For indomethacin, 42.3 mM SPC or 39.8 mM PCG_622_ LUVs were titrated into a 50.0 µM solution of indomethacin in matched buffer. For bexarotene, 6.00 mM SPC or 5.21 mM PCG_622_ LUVs were titrated into 30.0 µM bexarotene. The heat evolution was then plotted against the actual concentration of the lipids inside of the titration cell (calculated by the MicroCal PEAQ-ITC Analysis Software v1.41 considering dilution and overflow) and fitted to a non-linear partition model:

(2) $$Q_{i}={\left[L\right]}^{0}V_{i}\Delta_{dil}H+n_{D}^{0}\Delta_{p}H\frac{K_{p}\gamma [L]}{1+K_{p}\gamma [L]}$$

where $Q_{i}$ is the total heat evolved after i injections, ${\left[L\right]}^{0}$ is the stock lipid concentration, $V_{i}$ is the total volume injected, $\Delta_{dil}H$ is the uncorrected enthalpy associated with dilution and any non-specific effect, $n_{D}^{0}$ is the initial number of moles of drug, $\Delta_{p}H$ is the enthalpy associated with drug partitioning, [L] is the actual lipid concentration at the *i*-th injection as provided by the MicroCal PEAQ-ITC Analysis Software v1.41 and $K_{p}$ and *γ* have the same meaning as above. Detailed results and control experiments are reported in the Supplementary Materials.

### 3.7. Fluorescence Polarisation Anisotropy Experiments

Membrane fluidity studies were conducted using PCG_662_ and PCG_226_ LUVs, and by incorporating DPH and TMA-DPH. The LUVs were prepared by thin lipid-film hydration, and the probes were directly added during the thin-film formation after the solubilisation of DPH and TMA-DPH at 11.6 mg/mL in chloroform and in DMSO, respectively. The obtained LUVs were separated from unincorporated probes by passage through a Sepharose^®^ CL-4B loaded (Sigma-Aldrich) column and then characterised by DLS and NMR spectroscopy, as described above. The tested molecules were solubilised in DMSO at 100-fold the final concentration to obtain 2% of DMSO by addition of 2 µL in the 200 µL final volume. Fluorescence anisotropy measurements were performed every 30 min for 3 h at 25 °C using a Synergy 2 microplate reader (Biotek, Colmar, France) equipped with the appropriate filters (λ_ex_ = 360/40 nm and λ_em_ = 460/40 nm). The excitation and emission wavelengths were set to 358 nm and 429 nm, respectively. The sample were excited with vertically polarised light, and fluorescence intensities were recorded with the analysing polariser oriented parallel (I_||_) and perpendicular (I_⊥_) to the excitation polariser. The anisotropy emission was calculated according to the equation [70]:

(3) *r* = (*I_||_ − I_⊥_*)/(*I_||_ +* 2*I_⊥_*)

The results were expressed in percentage using the anisotropy of the samples without molecules (only 2% DMSO) as 100%.

### 3.8. Aβ Peptides Aggregation Kinetic Assay (ThT, Thioflavin T Fluorescence)

10 µM of Aβ peptides (Aβ_1-42_ or oG37C) were incubated in 96-well dark plates with ThT, at 25 °C in the presence or absence of liposomes (10 or 100 µM) and of tested compounds in Phosphate-buffered Saline (PBS) buffer (pH 7.4). By considering the reported oligomeric inhibiting potency (IC_50_) of indomethacin (958 µM), and ThT (122.19 ± 32.97 µM) [41], a ThT concentration of 20 µM was chosen to avoid any inhibiting effects specific to the ThT. Molecules were assayed at the following concentrations to bracket their IC_50_: 0.1, 1 and 10 µM for CSB; 10, 100 and 1000 µM for indomethacin. Bexarotene was assessed at 0.2, 2 and 20 µM [36]. To avoid the risk of the precipitation of compounds in stock or working solutions, BX and CSB were dissolved at 10 mM and IND at 50 mM in DMSO, then diluted in the same solvent and added directly to microplate wells to obtain the desired concentration with a normalised DMSO concentration of 2% (*v*/*v*) in all wells. Fluorescence measurements (λ_exc_ = 440 nm and λ_em_ = 484 nm) were made with an Infinite M200 microplate reader (Tecan, Männedorf, Switzerland). Data were collected as the average of 25 flashes each 10 min with 20 s of 1 mm orbital agitation before each run. Data are represented after blank subtraction; each representation combines the triplicate of an experiment.

### 3.9. Liposomes Leakage Assay (LLA)

Carboxyfluorescein (CF)-loaded LUVs were prepared by thin lipid-film hydration (see above). The hydration step was carried out with a 70 mM CF solution prepared in PBS buffer (pH adjusted at 7.4). The obtained LUVs were separated from unincorporated CF by passage through a Sepharose^®^ CL-4B loaded (Sigma-Aldrich) column using PBS buffer as eluent. The LUVs were characterised by DLS and the lipids were quantified by NMR spectroscopy as described above. The dequenching of the CF fluorescence was measured using a Synergy 2 microplate reader (Biotek, Colmar, France) equipped with the appropriate filters (λ_ex_ = 485/20 nm and λ_em_ = 528/20 nm). CF release assay was performed in a final volume of 100 μL, using 10 µM of LUVs. The fluorescence was recorded immediately (*F_0_*) and for 4 h at 25 °C. It was compared to that measured at the end of the experiment after the addition of 2 μL of 20% Triton X-100 solution to achieve complete liposome leakage (*F_max_*). To validate the LLA results, the sensibility rate R = *F_max_*/*F*_0_ was calculated, and it had to be greater than 6 to perform studies. Then, the percentage of CF release was calculated according to the following equation [71]:

(4) $$\%\;CF_{leakage}\;(t)=100\times \frac{\left(F_{t}-F_{0}\right)}{\left(F_{max}-F_{0}\right)}$$

where *F_t_* was the fluorescence intensity at time *t*, *F*_0_ the initial fluorescence intensity, and *F_max_* the final fluorescence intensity after addition of Triton X-100.

The data of LLA were plotted with GraphPad^®^ Prism statistical software (version 6.01, GraphPad^®^ Software, La Jolla, CA, USA). Comparison at references values (oG37c alone) were performed with a Mann–Whitney test. Differences were considered significant when the associated *p* value was below 0.05.

### 3.10. Attenuated Total Reflectance-Fourier Transform InfraRed Spectroscopy (ATR-FTIR)

Samples were incubated overnight at 25 °C before FTIR experiments to reach the plateau of the aggregation process. 3 µL of each sample was loaded on a germanium crystal and dried with a stream of dry nitrogen. ATR-FTIR spectra were recorded on a Nicolet IS50 FTIR spectrometer (Thermo Fisher Scientific, San Jose, CA, USA) equipped with DLaTG (Deuterated Lanthanum α-alanine-doped TriGlycine sulphate) and MCT (Mercury Cadmium Telluride) detectors with a spectral resolution of 2 cm^−1^. 128 interferograms were co-added after a 2 min acquisition period. All FTIR experiments were performed in a thermostatically controlled room at 25 °C. For signal processing, after blank subtraction, all spectra were fitted using OriginPro^®^ 8.5.1. The deconvolution of the amide I band of the spectra was performed using a Fast Fourier Transform (FFT) with low-pass filtering. The value of cutting frequency was determined by FFT of typical experimental FTIR spectra to remove high frequency contribution significantly larger than the typical amide frequency. The Voigt function type option was selected to consider two kinds of contributions: Lorentzian and Gaussian.

## 4. Conclusions

The aim of this work was to develop a set of simple orthogonal analytical methods (UV and fluorescence spectrophotometry, ATR-FTIR, ITC) to allow for the description of the interactions of small molecules with the Aβ peptide and biomembranes at an early stage of development. From results obtained on known aggregation modulators, bexarotene, Chicago sky blue and indomethacin, the interest of the proposed biomimetic multiparametric assay has been established. It could be transposed to identify original leads for the potential treatment of AD. Based on spectroscopy and spectrophotometry, those methods only require small amounts of compounds, which are indispensable at the drug discovery level. Thanks to the complementarity of the assays, it was possible to obtain crucial experimental data to describe the interactions between the molecule, the peptide and the membrane, even for molecules like CSB. Indeed, with compounds such as CSB that may induce artifacts in some type of spectroscopic experiments, the multiparametric workflow has helped with alleviating doubts thanks to multiple experiments with correlated results. It was already possible to differentiate action mechanisms of molecules in the absence or in the presence of membranes, confirming the need for the LUVs in the drug characterisation assays. Such a physico-chemical approach appeared to be well suited to drug discovery. It would be an undeniable asset for the rapid characterisation of compounds of therapeutic interest for Alzheimer’s disease. It has also paved the way for the combination of different molecules and targeted stages of amyloid assembly in a biomimetics context. This strategy could be used routinely to search for new modulators of the Aβ peptide. It could also be adapted and transposed to the search for modulators of other amyloids such as tau protein.

## Data Availability

Data are contained within the article and supplementary materials.

## Supplementary Materials

The following supporting information can be downloaded at: https://www.mdpi.com/article/10.3390/ijms242316982/s1. References [27,72] are cited in the supplementary materials.

## Abbreviations

Aβ: Amyloid β; AD: Alzheimer’s disease; ATR-FTIR: Attenuated Total Reflectance-Fourier Transform InfraRed; CF: bexarotene; BX: carboxyfluorescein; CHOL: cholesterol; CSB: Chicago sky blue 6B; DPH: 1,6-diphenyl-1,3,5-hexatriene; DOPG: 1,2-dioleoyl-sn-glycero-3-phosphoglycerol; DLS: dynamic light scattering; DMSO: dimethyl sulfoxide; Kp: partition coefficient; LLA: liposome leakage assay; LUVs: large unilamellar vesicles; PCG_622_: SPC:cholesterol:1,2-dioleoyl-sn-glycero-3-phosphoglycerol in a molar ratio of 6:2:2; PCG_226_: SPC:cholesterol:1,2-dioleoyl-sn-glycero-3-phosphoglycerol in a molar ratio of 2:2:6; PdI: Polydispersity Index; SPC: soybean phosphatidylcholine; ThT: thioflavine T; TMA-DPH: 1-(4-trimethylammoniumphenyl)-6-phenyl-1,3,5-hexatriene p-toluenesulfonate; TMSP: 3-(trimethylsilyl) propionic-2,2,3,3-d4 acid.
