# Supplementary material for: A Biomimetic Multiparametric Assay to Characterise Anti-Amyloid Drugs
Source: Int J Mol Sci. 2023 Nov 30;24(23):16982. doi: 10.3390/ijms242316982 (PMC10707238; doi:10.3390/ijms242316982)
Supplement: Supplementary file 1 [file ijms-24-16982-s001.zip › ijms-2712827-supplementary.pdf]

A biomimetic multiparametric assay to characterize anti amyloid drugs

Willy Smeralda[1#], Marc Since[1*], Sophie Corvaisier[1], Dimitri Fayolle[1], Julien Cardin[2], Sylvain Duprey[2], Jean-Pierre Jourdan[1,3], Christophe Cullin[4], Aurélie Malzert-Freon[1*]

[1]Normandie Univ, UNICAEN, CERMN, Boulevard Becquerel, 14032 Caen Cedex, France

[2]NIMPH Team, CIMAP Normandie Univ, ENSICAEN, UNICAEN, UMR6252 CNRS, CEA, 6 Bd du Maréchal Juin, 14050 Caen Cedex, France

[3]Pharmacie à Usage Intérieur, Centre Hospitalier de Vire, Vire, Normandie, FR 14504.

[4]CBMN, CNRS UMR 5248, Univ. Bordeaux, 33600 Pessac, France

Present Address:

[#]Laboratoire de Biophysique Moléculaire aux Interfaces, Gembloux Agro-Bio Tech, Université de Liège, 2 Passage des Déportés, B-5030 Gembloux, Belgium

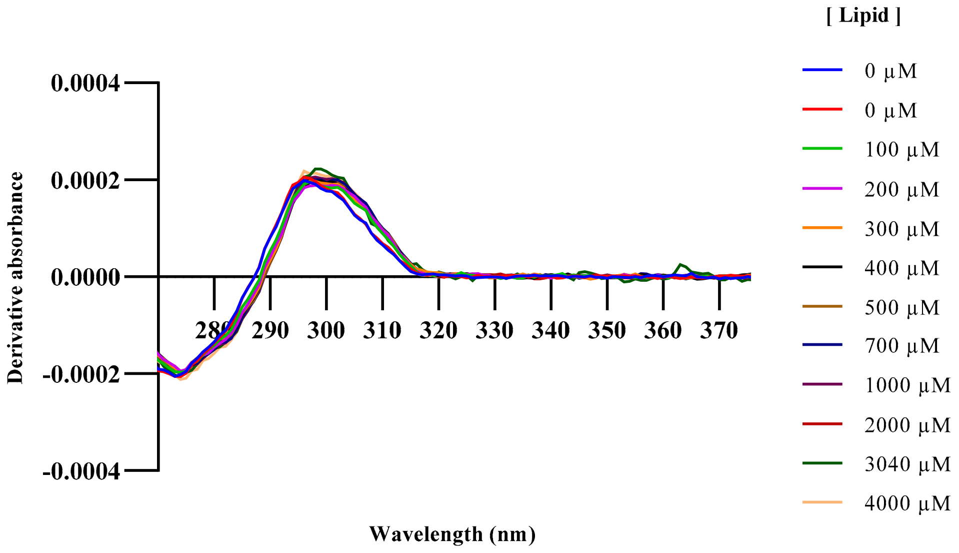

Figure S1. Second-derivative spectra of bexarotene (15 µM) incubated for 1h in the presence of increasing concentrations of lipids (PCG622-based LUVs: 0 to 4 mM) in Hepes buffer (pH 7.4) at 37°C.

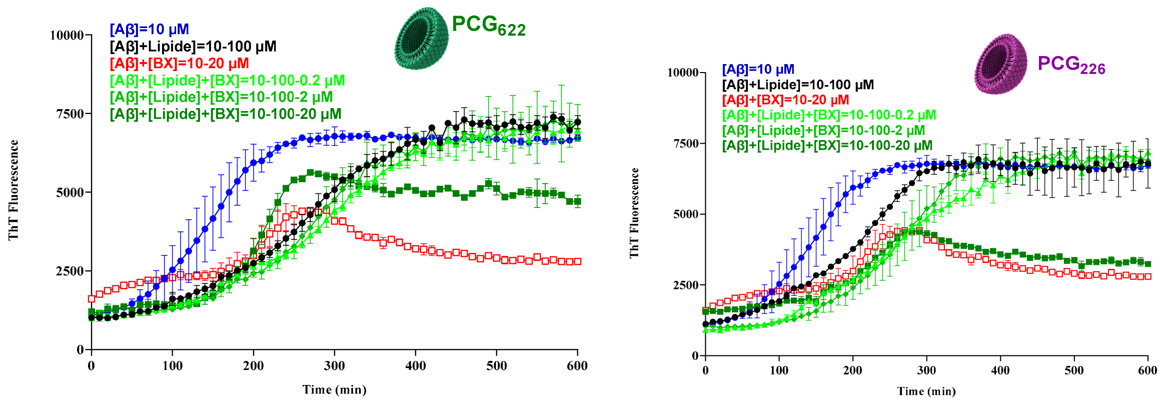

Figure S2. BX with 100 µM lipids. Kinetics of ThT fluorescence (20 µM) monitored for $A\beta_{1-42}$ alone 10 µM (blue dots), $A\beta_{1-42}$/bexarotene 20 µM (empty red squares), $A\beta_{1-42}$/PCG-based LUVs 100 µM (dark dots), $A\beta_{1-42}$/PCG-based LUVs/bexarotene 0.2 (light green triangle), 2 (green diamond), 20 µM (filled dark green squares). The error bars represent the standard deviation of 3 samples.

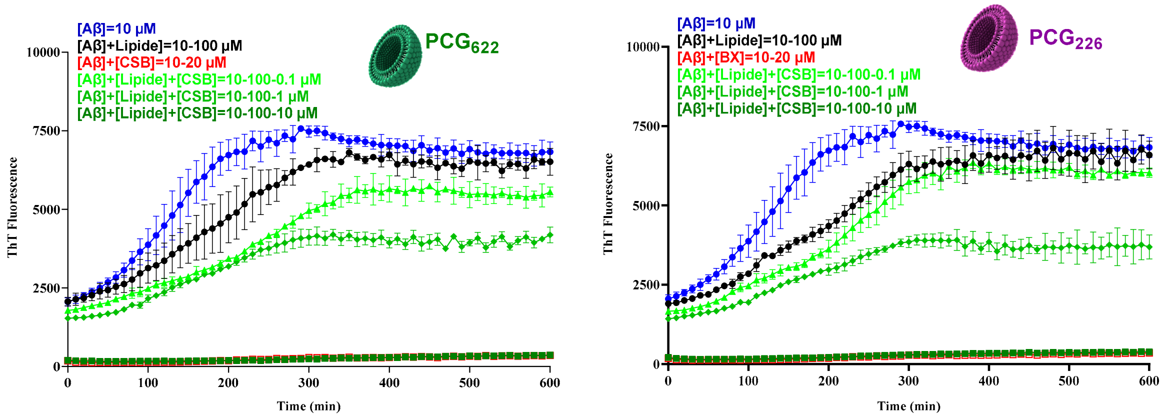

Figure S3. CSB with 100 µM lipids. Kinetics of ThT fluorescence (20 µM) monitored for $A\beta_{1-42}$ alone 10 µM (blue dots), $A\beta_{1-42}$/CSB 10 µM (empty red squares), $A\beta_{1-42}$/PCG-based LUVs 100 µM (dark dots), $A\beta_{1-42}$/PCG-based LUVs/CSB 0.1 (light green triangle), 1 (green diamond), 10 µM (filled dark green squares). The error bars represent the standard deviation of 3 samples.

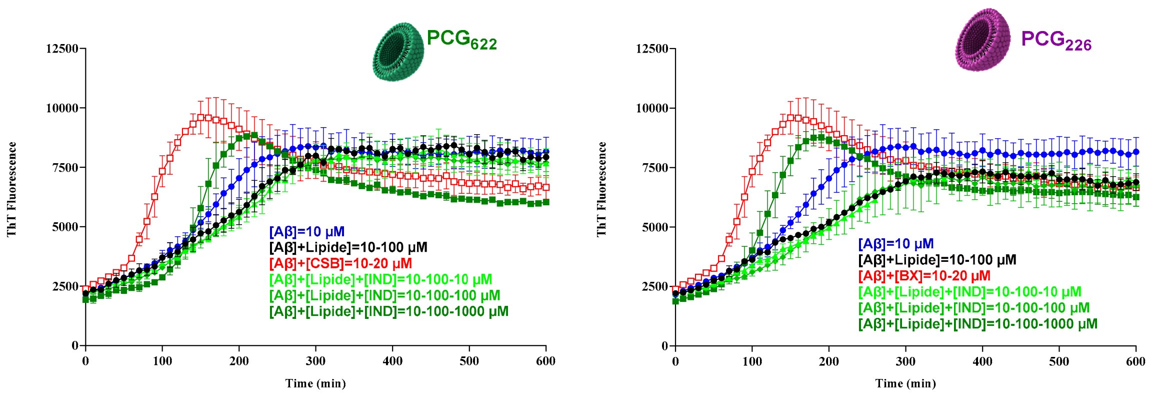

Figure S4. IND with 100 µM lipids. Kinetics of ThT fluorescence (20 µM) monitored for $A\beta_{1-42}$ alone 10 µM (blue dots), $A\beta_{1-42}$/CSB 10 µM (empty red squares), $A\beta_{1-42}$/PCG-based LUVs 100 µM (dark dots), $A\beta_{1-42}$/PCG-based LUVs/IND 10 (light green triangle), 100 (green diamond), 1000 µM (filled dark green squares). The error bars represent the standard deviation of 3 samples.

Table S1. Granulometric measurements of LUVs made of PCG_{226} (10 µM), expressed in terms of diameter by volume (nm), peak percentage of each population and PdI (polydispersity index), determined immediately after formulation, or after incubation at 25°C for 24h, alone, or in

presence of oG37C (10 µM) or/and **A.** bexarotene (BX, 20 µM), **B.** chicago sky blue 6B (CSB, 10 µM), **C.** indomethacin (IND, 1 mM).

A. Bexarotene (BX, 20 µM)

Interactome	Ø 1 (nm)	%	Ø 2 (nm)	%	Ø 3 (nm)	%	PdI
PCG_{226}, t_0	140.3 ± 1.3	100 ± 0.0					0.052 ± 0.013
PCG_{226}, t_{24}	148.9 ± 1.7	100 ± 0.0					0.047 ± 0.011
PCG_{226}, t_{24} + BX	153.4 ± 7.2	81.9 ± 10.3	3732.7 ± 2589.8	18.1 ± 10.3			0.331 ± 0.125
PCG_{226}, t_{24} + oG37C	159.4 ± 13.7	14.8 ± 2.2	1102.3 ± 30.7	75.0 ± 6.2	5058.3 ± 248.6	10.2 ± 4.0	0.600 ± 0.151
PCG_{226}, t_{24} + oG37C + BX	214.5 ± 2.2	15.9 ± 2.0	1237.3 ± 89.8	73.6 ± 2.5	5079.7 ± 204.5	10.5 ± 0.5	0.790 ± 0.052

B. Chicago sky blue 6B (CSB, 10 µM)

Interactome	Ø 1 (nm)	%	Ø 2 (nm)	%	Ø3 (nm)	%	PdI
PCG_{226}, t_0	135.5 ± 1.3	100 ± 0.0					0.051 ± 0.016
PCG_{226}, t_{24}	141.6 ± 0.8	100 ± 0.0					0.071 ± 0.008
PCG_{226}, t_{24} + CSB	140.6 ± 0.9	100 ± 0.0					0.086 ± 0.014
PCG_{226}, t_{24} + oG37C	176.8 ± 16.3	23.6 ± 3.6	1524.3 ± 237.5	73.1 ± 5.2	3205.3 ± 2782.7	3.4 ± 3.4	0.778 ± 0.024
PCG_{226}, t_{24} + oG37C + CSB	141.1 ± 4.3	49.1 ± 14.1	1488.7 ± 15.9	44.3 ± 25.5	1487.0 ± 2575.6	6.6 ± 11.5	0.383 ± 0.037

C. Indomethacin (IND, 1 mM).

Interactome	Ø 1 (nm)	%	Ø 2 (nm)	%	Ø3 (nm)	%	PdI
PCG_{226}, t_0	135.5 ± 1.3	100 ± 0.0					0.051 ± 0.016
PCG_{226}, t_{24}	141.6 ± 0.8	100 ± 0.0					0.071 ± 0.008
PCG_{226}, t_{24} + IND	142.8 ± 1.7	100 ± 0.0					0.073 ± 0.013
PCG_{226}, t_{24} + oG37C	176.8 ± 16.3	23.6 ± 3.6	1524.3 ± 237.5	73.1 ± 5.2	3205.3 ± 2782.7	3.4 ± 3.4	0.778 ± 0.024
PCG_{226}, t_{24} + oG37C + IND	49.6 ± 8.9	46.6 ± 5.3	250.5 ± 79.6	14.3 ± 3.9	1513.0 ± 16.1	36.0 ± 11.6	0.763 ± 0.094

Membrane binding by ITC: control experiments and additional information

Titration of the drug solution into liposomes

The titration described in the main paper involves the simultaneous determination of K_p and $\Delta_p H$ in the same fit. To ensure the consistency of our fit, we independently determined $\Delta_p H$ in a separate experiment for one titration. To do so, 200 µM indomethacin in matched buffer was

titrated into 6.00 mM SPC LUVs so that the lipids were always in large excess (approximately 2500 :1 at the first injection and 100 :1 at the last injection, one dummy 0.4 µL injection followed by 18x2 µL injections) and virtually all of the drug partitioned in the membrane. As expected, each injection resulted in a nearly identical heat release, confirming that membrane saturation never occurred to a significant extent. Linear regression of the total heat released at the ith injection Q_i against the number of moles added afforded the uncorrected enthalpy ΔH of the binding process. A control experiment without liposomes was performed (the test compound was titrated into matched buffer) and a similar linear regression gave the enthalpy associated with dilution $\Delta_{dil}H$. The difference afforded the partition enthalpy $\Delta_p H$.

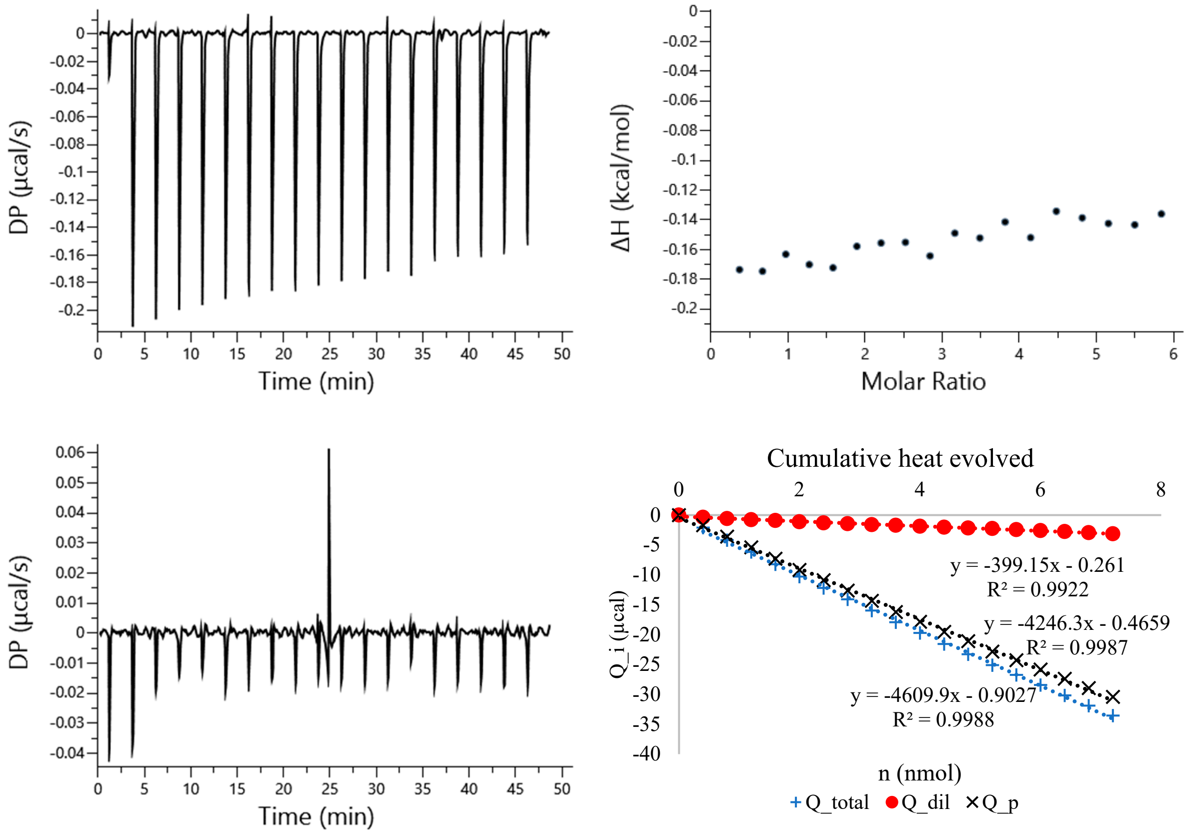

Figure S5. Thermograms of the titration of 200 µM indomethacin into 6.00 mM SPC LUVs. Top
left: raw data after baseline correction; top right: integrated heat evolution; bottom left: control experiment without LUVs after baseline correction; bottom right: linear regression.

Indomethacin

$\Delta H = -19.3 \pm 0.1$ kJ/mol

$\Delta_{dil}H = -1.67 \pm 0.04$ kJ/mol

$\Delta_p H = -17.6 \pm 0.1$ kJ/mol (Matos et al 2004 [72] found -17.2 kJ/mol)

Bexarotene was not soluble above 30 µM and the heat evolution when 30.0 µM bexarotene was titrated into liposomes was too low to determine ΔH.

Titration of the liposomes into the drug solution

To determine K_p, a reverse titration was performed. For indomethacin, 42.3 mM SPC LUVs or 39.8 mM PCG_{622} LUVs were titrated into a 50.0 µM solution of indomethacin in matched buffer. For bexarotene, 6.00 mM SPC or 5.21 mM anionic LUVs were titrated into 30.0 µM bexarotene. The heat evolution was then plotted against the actual concentration of the lipids inside of the titration cell (calculated by the ITC software considering dilution and overflow) and fitted to a non-linear partition model:

$$Q_i = [L]^0 V_i \Delta_{dil} H + n_D^0 \Delta_p H \frac{K_p \gamma [L]}{1 + K_p \gamma [\mathrm{L}]}$$

Where Q_i is the total heat evolved after i injections, $[L]^0$ is the stock lipid concentration, V_i is the total volume injected, $\Delta_{dil}H$ is the uncorrected enthalpy associated with dilution and any non-specific effect, n_D^0 is the initial number of moles of drug, $\Delta_p H$ is the enthalpy associated with drug partitioning, K_p is the dimensionless partition coefficient, $\gamma = 0.70$ L/mol is the molar volume of SPC membranes, and $[L]$ is the actual lipid concentration at the i-th injection as provided by the ITC software. The first member of this equation represents the linear heat evolution related to dilution. The second term represents a partition equilibrium. This equation was discussed elsewhere (Matos et al 2004 [72], Smeralda et al 2019 [27]). An iterative non-linear fit was performed by minimizing the sum of squared residuals with the solver module of Microsoft Excel.

The dilution heat term encompasses any non-specific effect linked with the mixing of the drug solution and the lipid solution, including slight composition mismatches. In particular, DMSO has an extremely high heat of dilution and very small mismatches can result in considerable heat evolution. Although the dilution heat was found to be extremely low in all fits ($|\Delta_{\mathrm{dil}}\mathrm{H}| \leq$ 10 cal/mol, i.e. at least 100 times lower than $\Delta_{\mathrm{p}}\mathrm{H}$), omitting this term from the fit would result in a significant fit error. Indeed, partitioning-induced heat releases were small in absolute value due to remarkably high drug dilution, and the cumulative effect of dilution heat, even very small individually, ended up being significant.

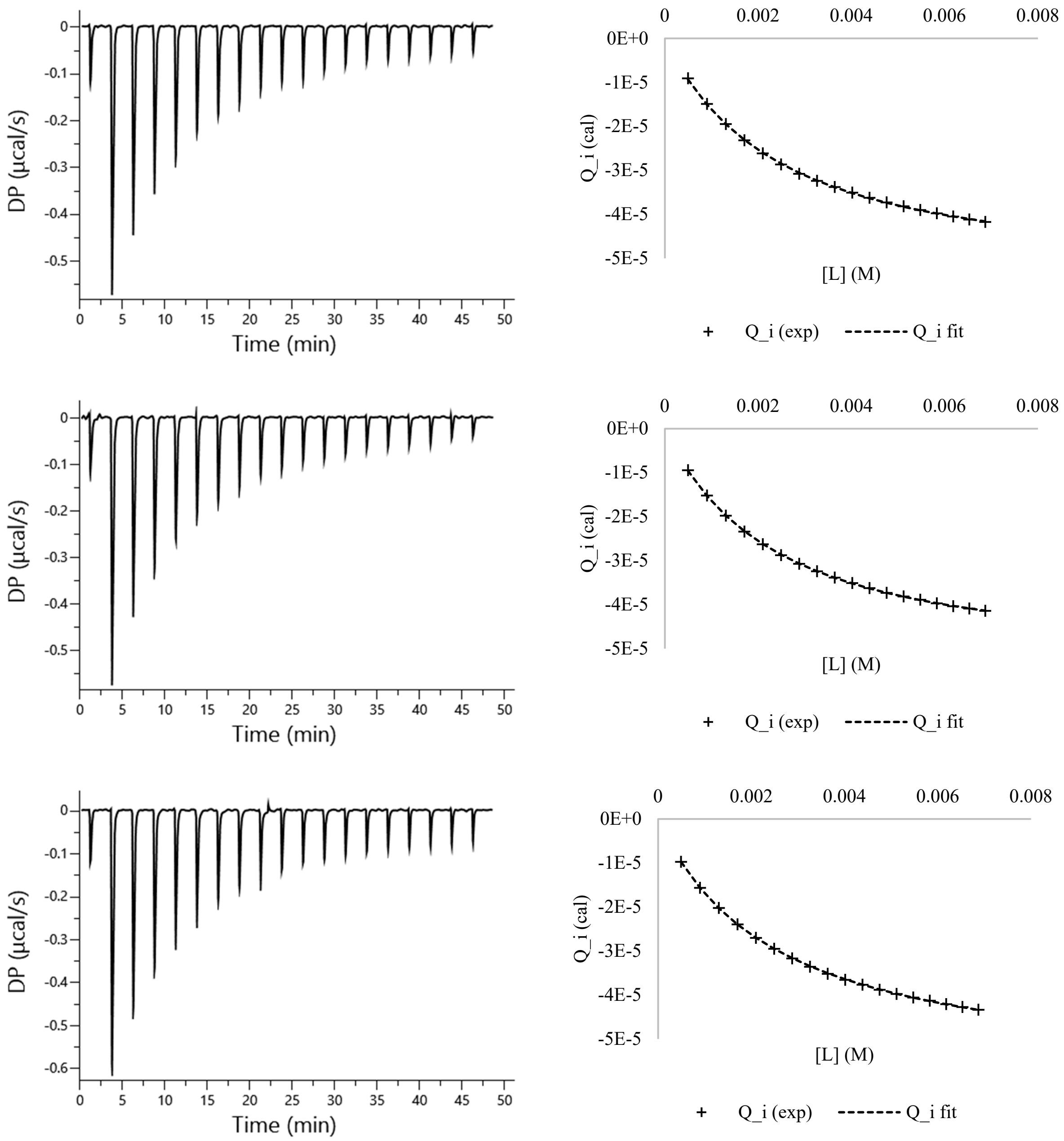

Figure S6. Titration of 6.00 mM SPC LUVs into 50.0 µM indomethacin (N = 3). Left: thermograms with baseline correction; right: cumulative plots with fits.

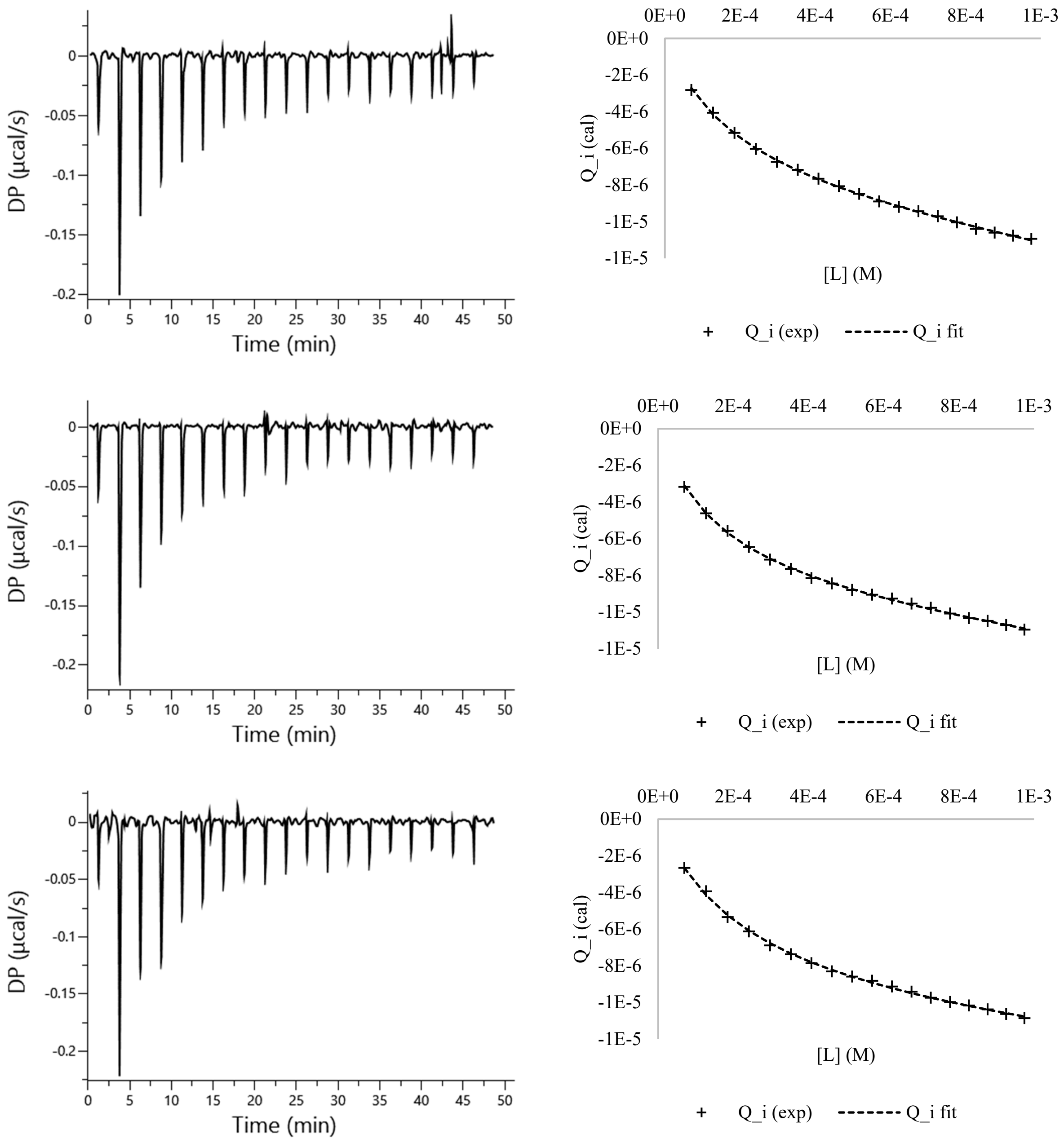

Figure S7. Titration of 6.00 mM SPC LUVs into 30.0 μM bexarotene (N = 3). Left: thermograms with baseline correction; right: cumulative plots with fits.

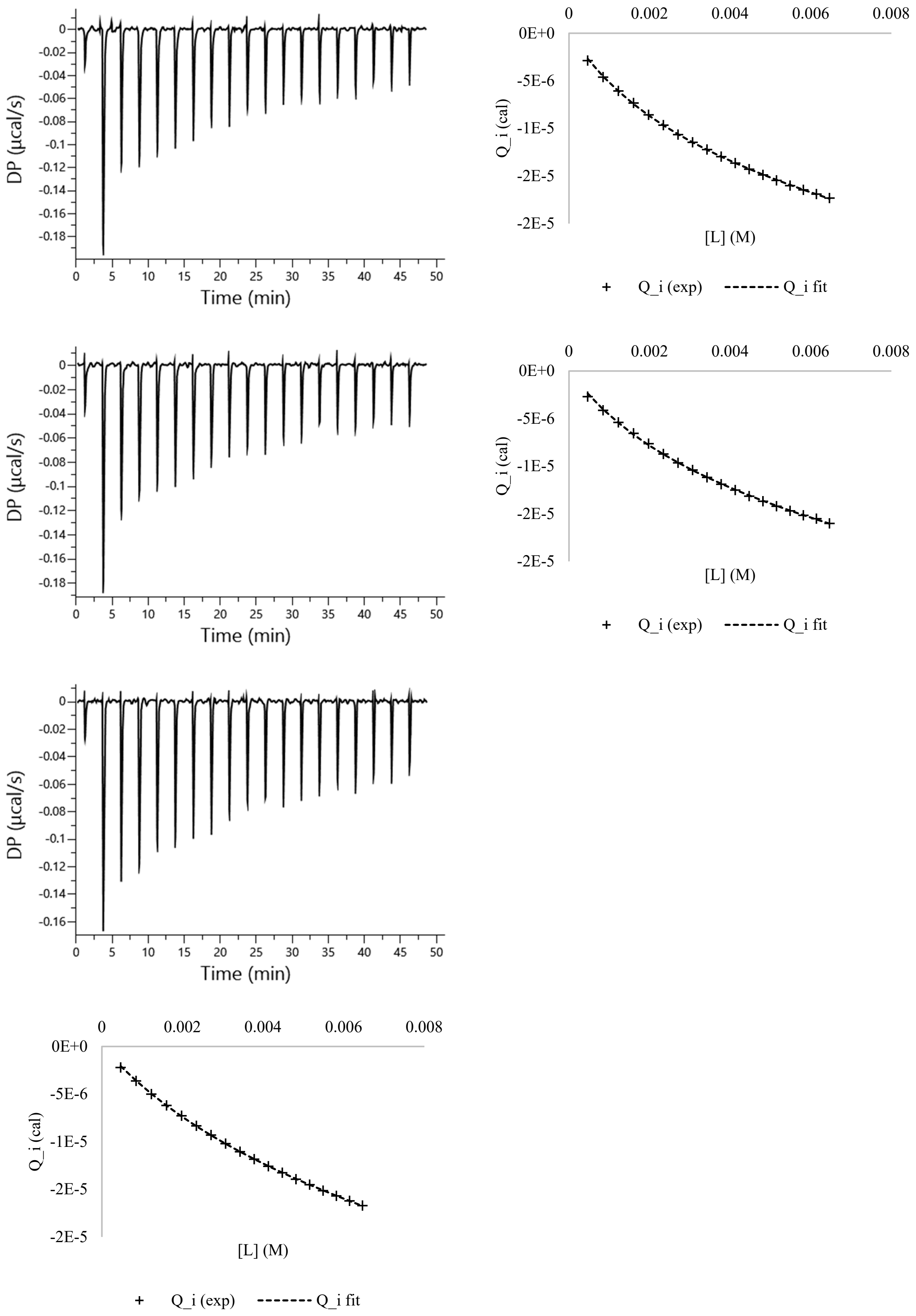

Figure S8. Titration of 39.8 mM PCG$_{622}$ LUVs into 50.0 µM indomethacin (N = 3). Left: thermograms with baseline correction; right: cumulative plots with fits.

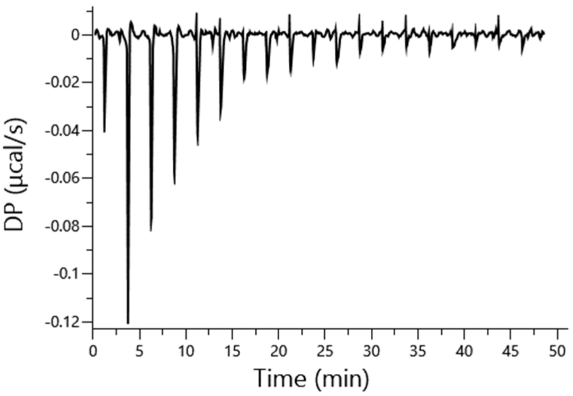
DP (µcal/s)
Time (min)

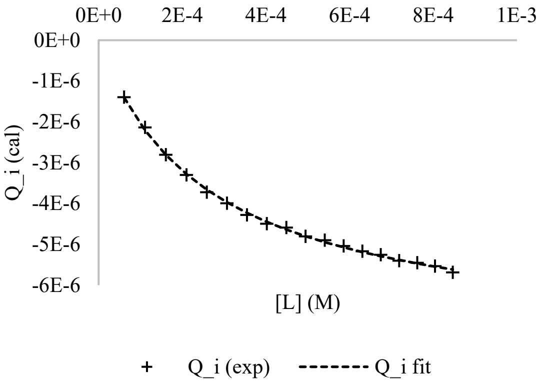
Q_i (cal)
[L] (M)
Q_i (exp)
Q_i fit

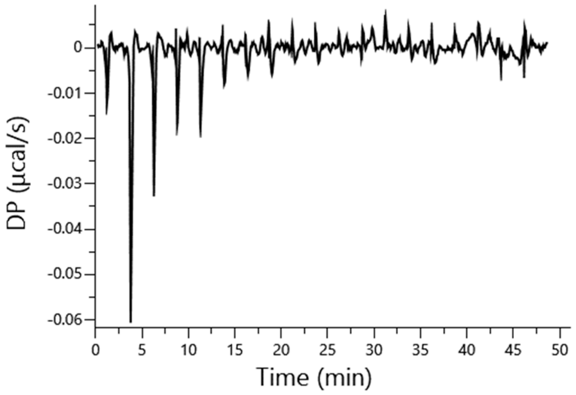
DP (µcal/s)
Time (min)

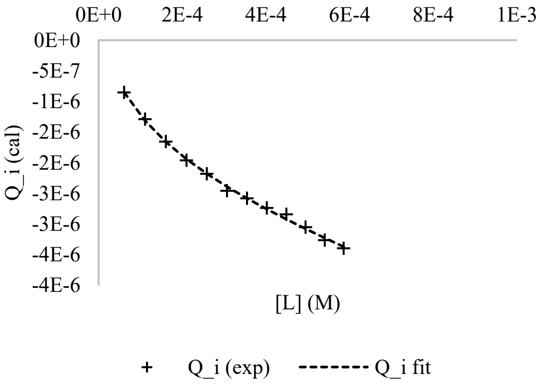
Q_i (cal)
[L] (M)
Q_i (exp)
Q_i fit

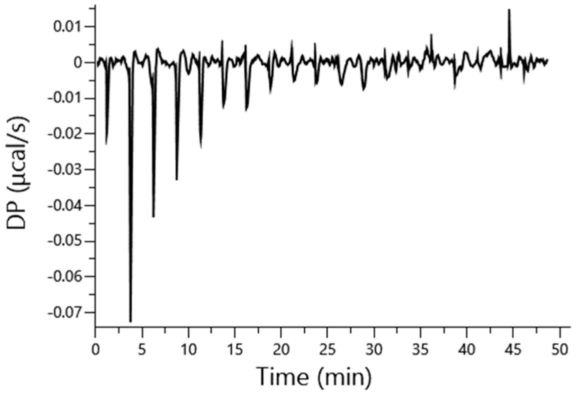
DP (µcal/s)
Time (min)

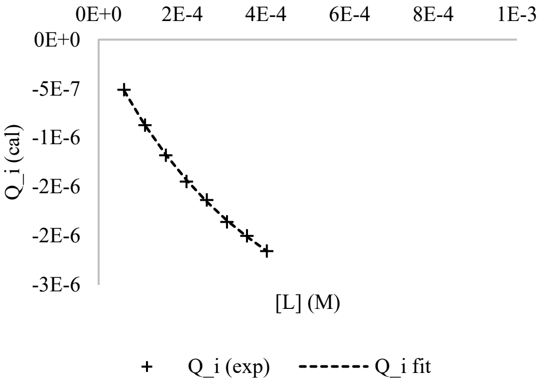
Q_i (cal)
[L] (M)
Q_i (exp)
Q_i fit

Figure S9. Titration of 5.23 mM PCG_{622} LUVs into 30.0 µM bexarotene (N = 3). Left: thermograms with baseline correction; right: cumulative plots with fits. Due to a very weak signal, analyses were truncated when no more useful signal could be interpreted.

Indomethacin and SPC LUVs: K_p = 561 ± 15, log (K_p) = 2.75, $\Delta_p H$ = -19.8 ± 0.2 kJ/mol

Bexarotene and SPC LUVs: K_p = 7300 ± 1100, log (K_p) = 3.86, $\Delta_p H$ = -5.8 ± 0.4 kJ/mol

Indomethacin and PCG_{622} LUVs: K_p = 360 ± 76, log (K_p) = 2.56, $\Delta_p H$ = -7.0 ± 0.7 kJ/mol

Bexarotene and PCG_{622} LUVs: K_p = 6000 ± 3000, log (K_p) = 3.78, $\Delta_p H$ = -3.2 ± 1.2 kJ/mol

Calculation of mol% bond of BX bond to lipids

$$mol\% = \frac{Q_{BX}^{lipid}}{Q_{BX}^{total}} \times 100 \qquad \text{(S1)}$$

Where $Q_{BX}^{total}$is the total BX quantity (mol) in well, Q_{BX}^{lipid}, the BX quantity (mol) bond to lipids.

$$Q_{BX}^{total} = [BX]_{aqueous} \times V_{total} \qquad \text{(S2)}$$

$$Q_{BX}^{lipid} = [BX]_{lipid} \times V_{lipid} \qquad \text{(S3)}$$

$$V_{lipid} = [lipid] \times V_{total} \times \gamma \qquad \text{(S4)}$$

$$[BX]_{lipid} = Kp \times [BX]_{aqueous} \qquad \text{(S5)}$$

Where $[BX]_{aqueous}$ is the BX concentration (M) in aqueous phase (considered constant), and V_{total}, the total solution volume (L), $[BX]_{lipid}$ is the BX concentration in lipids, V_{lipid} is the total solution volume of lipids (L), $[lipid]$ is the lipid concentration (M), γ, the molar volume of lipids (0,7 mol.L^{-1}), Kp is the partition coefficient obtained using ITC.

From (S1), (S2), (S3), (S4), (S5)

$$mol\% = [lipid] \times \gamma \times Kp \times 100 \qquad \text{(S6)}$$
